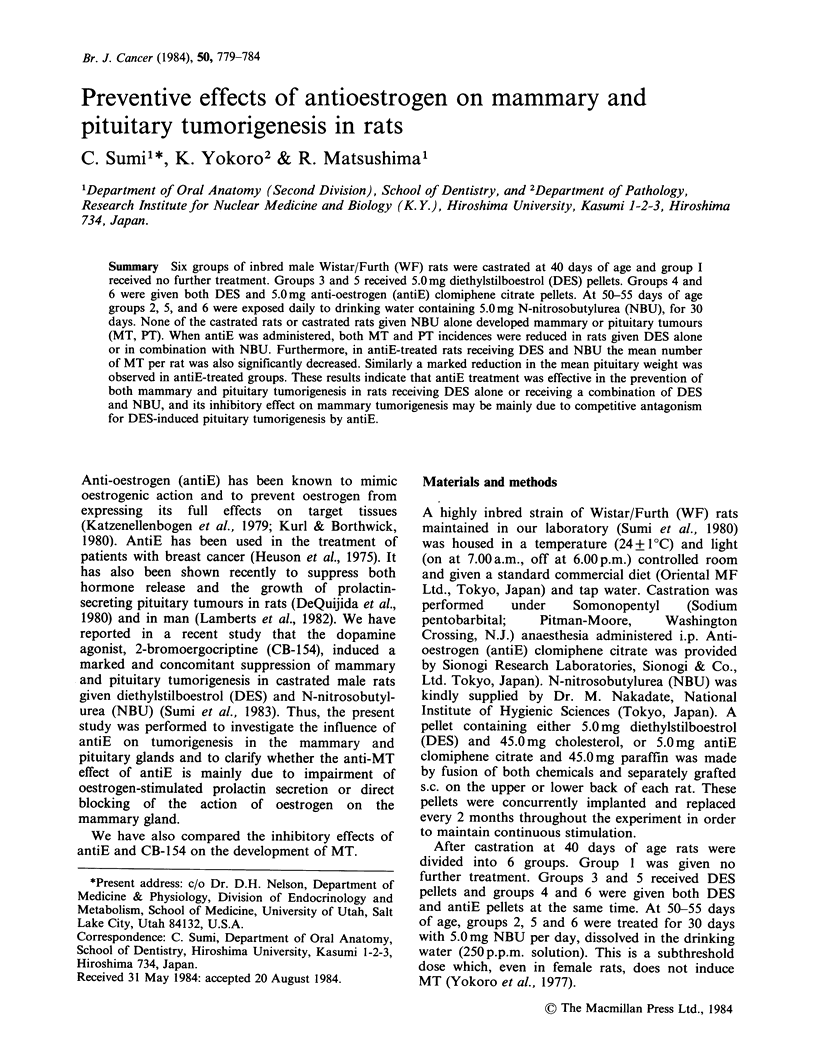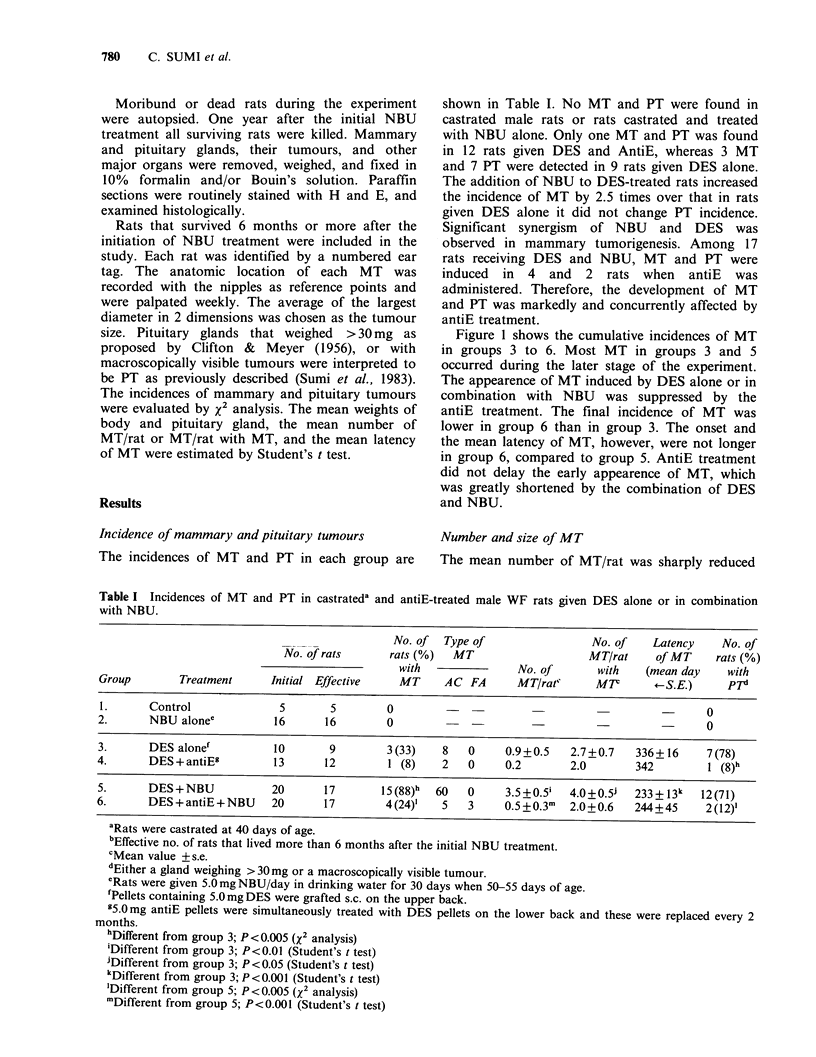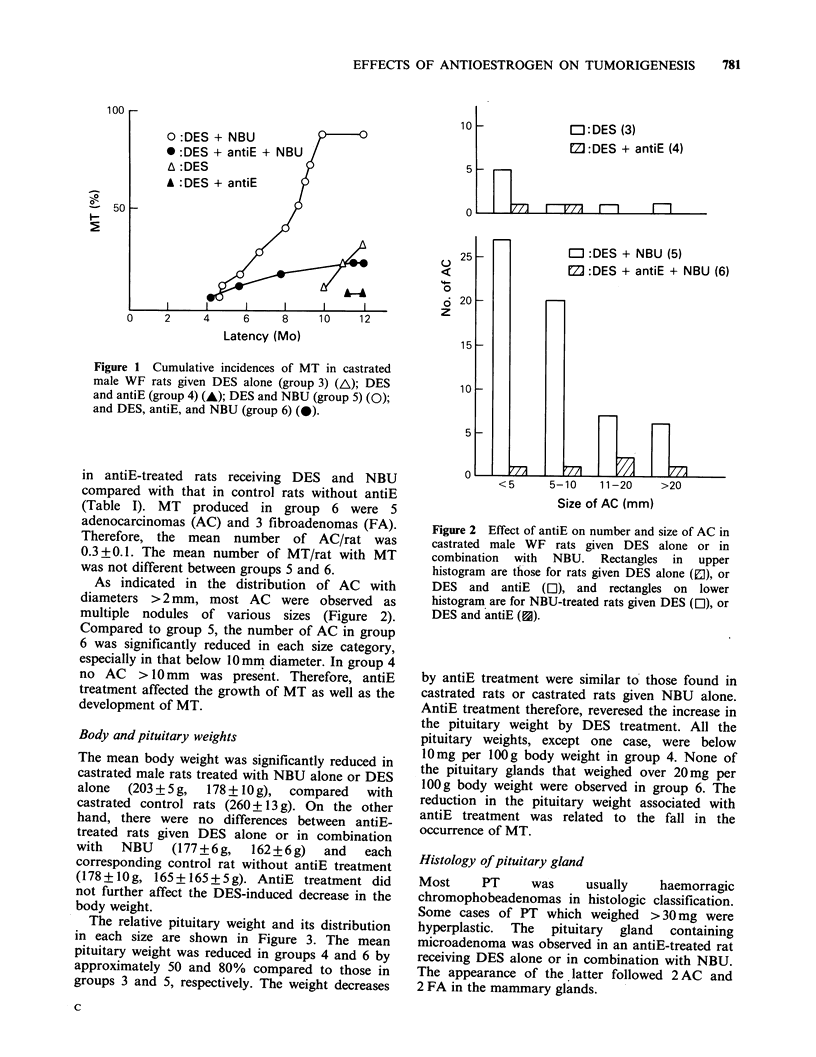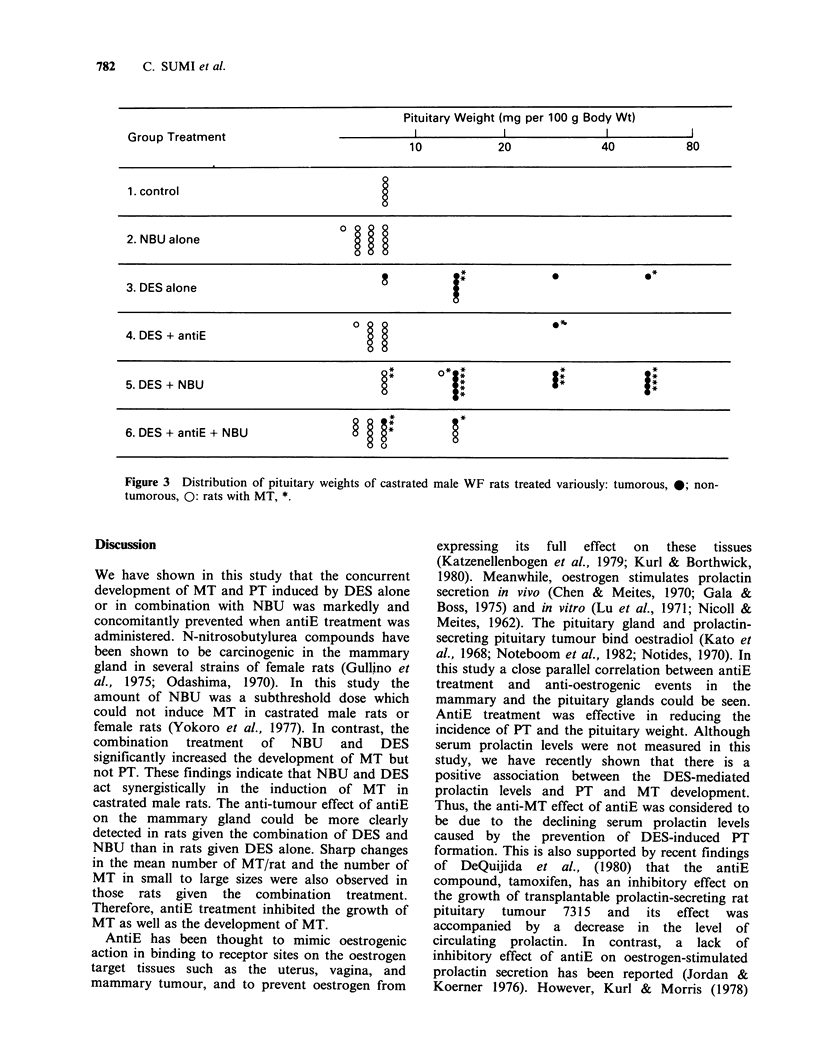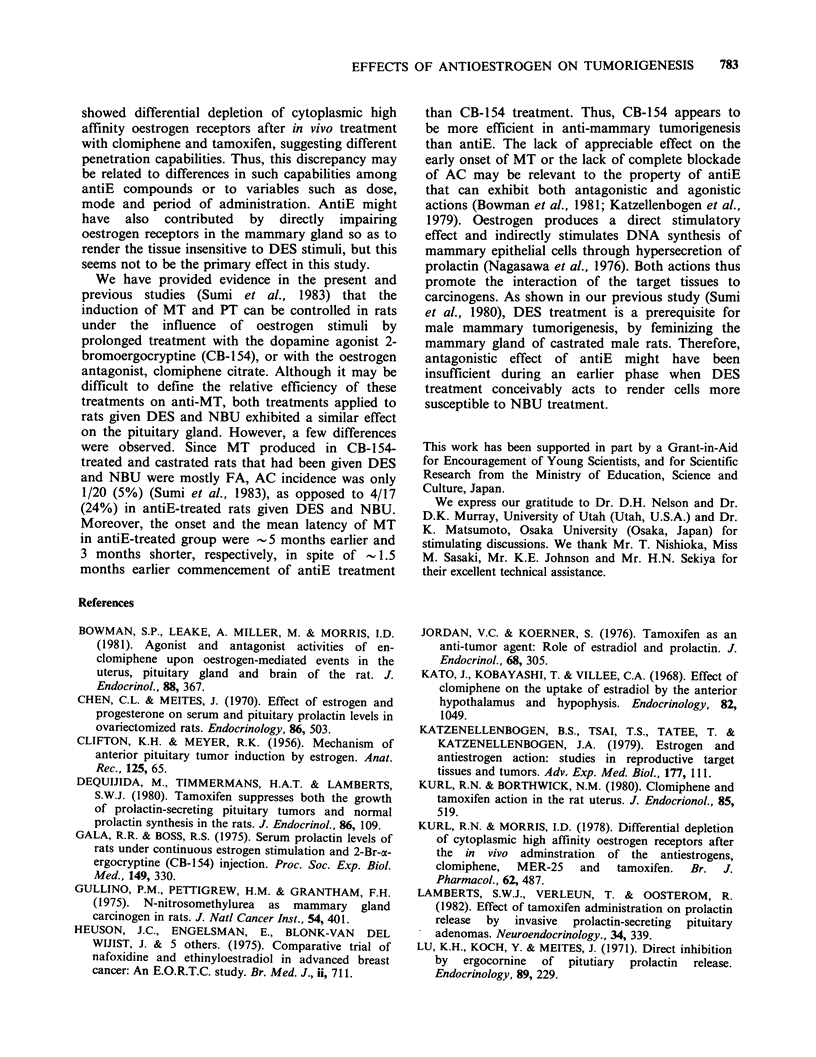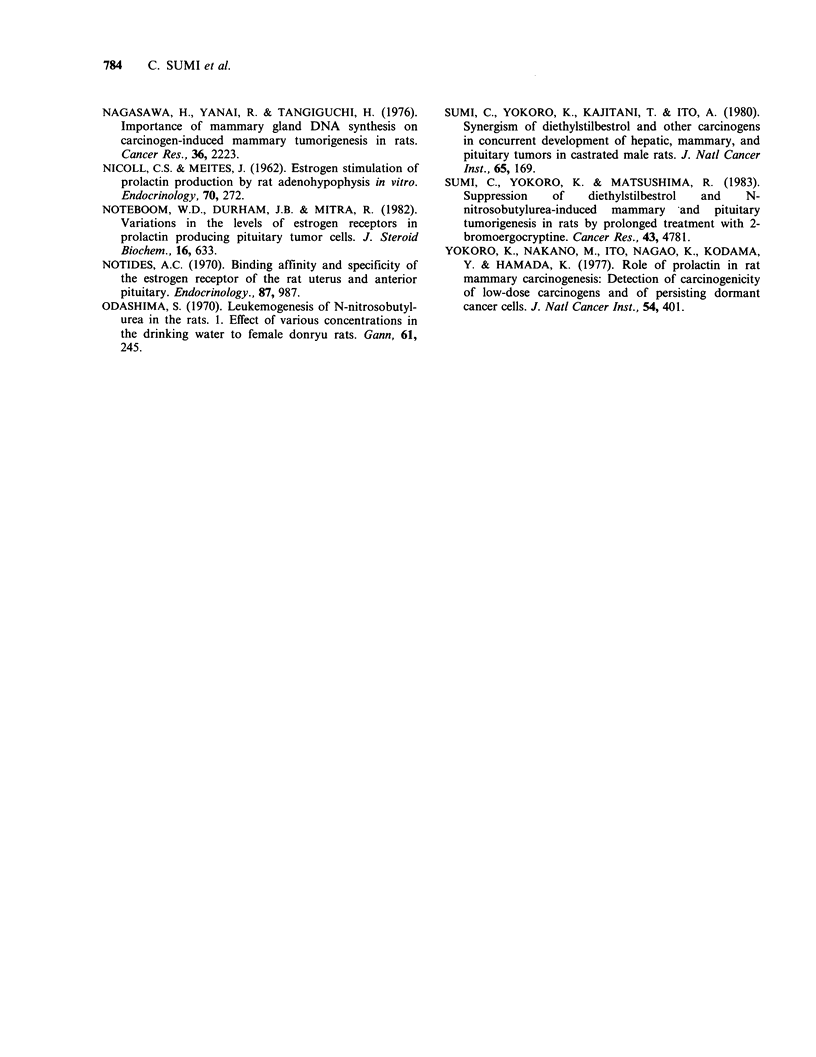# Preventive effects of antioestrogen on mammary and pituitary tumorigenesis in rats.

**DOI:** 10.1038/bjc.1984.256

**Published:** 1984-12

**Authors:** C. Sumi, K. Yokoro, R. Matsushima

## Abstract

Six groups of inbred male Wistar/Furth (WF) rats were castrated at 40 days of age and group I received no further treatment. Groups 3 and 5 received 5.0 mg diethylstilboestrol (DES) pellets. Groups 4 and 6 were given both DES and 5.0 mg anti-oestrogen (antiE) clomiphene citrate pellets. At 50-55 days of age groups 2, 5, and 6 were exposed daily to drinking water containing 5.0 mg N-nitrosobutylurea (NBU), for 30 days. None of the castrated rats given NBU alone developed mammary or pituitary tumours (MT, PT). When antiE was administered, both MT and PT incidences were reduced in rats given DES alone or in combination with NBU. Furthermore, in antiE-treated rats receiving DES and NBU the mean number of MT per rat was also significantly decreased. Similarly a marked reduction in the mean pituitary weight was observed in antiE-treated groups. These results indicate that antiE treatment was effective in the prevention of both mammary and pituitary tumorigenesis in rats receiving DES alone or receiving a combination of DES and NBU, and its inhibitory effect on mammary tumorigenesis may be mainly due to competitive antagonism for DES-induced pituitary tumorigenesis by antiE.


Br. J. Cancer (1984), **50**, 779–784

### Preventive effects of antioestrogen on mammary and pituitary tumorigenesis in rats

C. Sumi[1]*, K. Yokoro[2] & R. Matsushima[1]

[1]*Department of Oral Anatomy (Second Division), School of Dentistry, and* [2]*Department of Pathology, Research Institute for Nuclear Medicine and Biology (K.Y.), Hiroshima University, Kasumi 1-2-3, Hiroshima 734, Japan.*

**Summary** Six groups of inbred male Wistar/Furth (WF) rats were castrated at 40 days of age and group I received no further treatment. Groups 3 and 5 received 5.0 mg diethylstilboestrol (DES) pellets. Groups 4 and 6 were given both DES and 5.0 mg anti-oestrogen (antiE) clomiphene citrate pellets. At 50–55 days of age groups 2, 5, and 6 were exposed daily to drinking water containing 5.0 mg N-nitrosobutylurea (NBU), for 30 days. None of the castrated rats or castrated rats given NBU alone developed mammary or pituitary tumours (MT, PT). When antiE was administered, both MT and PT incidences were reduced in rats given DES alone or in combination with NBU. Furthermore, in antiE-treated rats receiving DES and NBU the mean number of MT per rat was also significantly decreased. Similarly a marked reduction in the mean pituitary weight was observed in antiE-treated groups. These results indicate that antiE treatment was effective in the prevention of both mammary and pituitary tumorigenesis in rats receiving DES alone or receiving a combination of DES and NBU, and its inhibitory effect on mammary tumorigenesis may be mainly due to competitive antagonism for DES-induced pituitary tumorigenesis by antiE.

Anti-oestrogen (antiE) has been known to mimic oestrogenic action and to prevent oestrogen from expressing its full effects on target tissues (Katzenellenbogen *et al.*, 1979; Kurl & Borthwick, 1980). AntiE has been used in the treatment of patients with breast cancer (Heuson *et al.*, 1975). It has also been shown recently to suppress both hormone release and the growth of prolactin-secreting pituitary tumours in rats (DeQuijida *et al.*, 1980) and in man (Lamberts *et al.*, 1982). We have reported in a recent study that the dopamine agonist, 2-bromoergocriptine (CB-154), induced a marked and concomitant suppression of mammary and pituitary tumorigenesis in castrated male rats given diethylstilboestrol (DES) and N-nitrosobutylurea (NBU) (Sumi *et al.*, 1983). Thus, the present study was performed to investigate the influence of antiE on tumorigenesis in the mammary and pituitary glands and to clarify whether the anti-MT effect of antiE is mainly due to impairment of oestrogen-stimulated prolactin secretion or direct blocking of the action of oestrogen on the mammary gland.

We have also compared the inhibitory effects of antiE and CB-154 on the development of MT.

*Present address: c/o Dr. D.H. Nelson, Department of Medicine & Physiology, Division of Endocrinology and Metabolism, School of Medicine, University of Utah, Salt Lake City, Utah 84132, U.S.A.
Correspondence: C. Sumi, Department of Oral Anatomy, School of Dentistry, Hiroshima University, Kasumi 1-2-3, Hiroshima 734, Japan.
Received 31 May 1984: accepted 20 August 1984.

### Materials and methods

A highly inbred strain of Wistar/Furth (WF) rats maintained in our laboratory (Sumi *et al.*, 1980) was housed in a temperature (24 ± 1°C) and light (on at 7.00 a.m., off at 6.00 p.m.) controlled room and given a standard commercial diet (Oriental MF Ltd., Tokyo, Japan) and tap water. Castration was performed under Somonopentyl (Sodium pentobarbital; Pitman-Moore, Washington Crossing, N.J.) anaesthesia administered i.p. Anti-oestrogen (antiE) clomiphene citrate was provided by Sionogi Research Laboratories, Sionogi & Co., Ltd. Tokyo, Japan). N-nitrosobutylurea (NBU) was kindly supplied by Dr. M. Nakadate, National Institute of Hygienic Sciences (Tokyo, Japan). A pellet containing either 5.0 mg diethylstilboestrol (DES) and 45.0 mg cholesterol, or 5.0 mg antiE clomiphene citrate and 45.0 mg paraffin was made by fusion of both chemicals and separately grafted s.c. on the upper or lower back of each rat. These pellets were concurrently implanted and replaced every 2 months throughout the experiment in order to maintain continuous stimulation.

After castration at 40 days of age rats were divided into 6 groups. Group 1 was given no further treatment. Groups 3 and 5 received DES pellets and groups 4 and 6 were given both DES and antiE pellets at the same time. At 50–55 days of age, groups 2, 5 and 6 were treated for 30 days with 5.0 mg NBU per day, dissolved in the drinking water (250 p.p.m. solution). This is a subthreshold dose which, even in female rats, does not induce MT (Yokoro *et al.*, 1977).

© The Macmillan Press Ltd., 1984

Moribund or dead rats during the experiment were autopsied. One year after the initial NBU treatment all surviving rats were killed. Mammary and pituitary glands, their tumours, and other major organs were removed, weighed, and fixed in 10% formalin and/or Bouin's solution. Paraffin sections were routinely stained with H and E, and examined histologically.

Rats that survived 6 months or more after the initiation of NBU treatment were included in the study. Each rat was identified by a numbered ear tag. The anatomic location of each MT was recorded with the nipples as reference points and were palpated weekly. The average of the largest diameter in 2 dimensions was chosen as the tumour size. Pituitary glands that weighed >30 mg as proposed by Clifton & Meyer (1956), or with macroscopically visible tumours were interpreted to be PT as previously described (Sumi *et al.*, 1983). The incidences of mammary and pituitary tumours were evaluated by $\chi^2$ analysis. The mean weights of body and pituitary gland, the mean number of MT/rat or MT/rat with MT, and the mean latency of MT were estimated by Student's *t* test.

### Results

### *Incidence of mammary and pituitary tumours*

The incidences of MT and PT in each group are shown in Table I. No MT and PT were found in castrated male rats or rats castrated and treated with NBU alone. Only one MT and PT was found in 12 rats given DES and AntiE, whereas 3 MT and 7 PT were detected in 9 rats given DES alone. The addition of NBU to DES-treated rats increased the incidence of MT by 2.5 times over that in rats given DES alone it did not change PT incidence. Significant synergism of NBU and DES was observed in mammary tumorigenesis. Among 17 rats receiving DES and NBU, MT and PT were induced in 4 and 2 rats when antiE was administered. Therefore, the development of MT and PT was markedly and concurrently affected by antiE treatment.

Figure 1 shows the cumulative incidences of MT in groups 3 to 6. Most MT in groups 3 and 5 occurred during the later stage of the experiment. The appearence of MT induced by DES alone or in combination with NBU was suppressed by the antiE treatment. The final incidence of MT was lower in group 6 than in group 3. The onset and the mean latency of MT, however, were not longer in group 6, compared to group 5. AntiE treatment did not delay the early appearence of MT, which was greatly shortened by the combination of DES and NBU.

### *Number and size of MT*

The mean number of MT/rat was sharply reduced

**Table I** Incidences of MT and PT in castrated[a] and antiE-treated male WF rats given DES alone or in combination with NBU.

| Group | Treatment | No. of rats: Initial | No. of rats: Effective | No. of rats (%) with MT | Type of MT: AC | Type of MT: FA | No. of MT/rat[c] | No. of MT/rat with MT[c] | Latency of MT (mean day ±S.E.) | No. of rats (%) with PT[d] |
|---|---|---|---|---|---|---|---|---|---|---|
| 1. | Control | 5 | 5 | 0 | — | — | — | — | — | 0 |
| 2. | NBU alone[e] | 16 | 16 | 0 | — | — | — | — | — | 0 |
| 3. | DES alone[f] | 10 | 9 | 3 (33) | 8 | 0 | 0.9±0.5 | 2.7±0.7 | 336±16 | 7 (78) |
| 4. | DES+antiE[g] | 13 | 12 | 1 (8) | 2 | 0 | 0.2 | 2.0 | 342 | 1 (8)[h] |
| 5. | DES+NBU | 20 | 17 | 15 (88)[h] | 60 | 0 | 3.5±0.5[i] | 4.0±0.5[j] | 233±13[k] | 12 (71) |
| 6. | DES+antiE+NBU | 20 | 17 | 4 (24)[l] | 5 | 3 | 0.5±0.3[m] | 2.0±0.6 | 244±45 | 2 (12)[l] |

[a]Rats were castrated at 40 days of age.
[b]Effective no. of rats that lived more than 6 months after the initial NBU treatment.
[c]Mean value ±s.e.
[d]Either a gland weighing >30 mg or a macroscopically visible tumour.
[e]Rats were given 5.0 mg NBU/day in drinking water for 30 days when 50–55 days of age.
[f]Pellets containing 5.0 mg DES were grafted s.c. on the upper back.
[g]5.0 mg antiE pellets were simultaneously treated with DES pellets on the lower back and these were replaced every 2 months.
[h]Different from group 3; $P<0.005$ ($\chi^2$ analysis)
[i]Different from group 3; $P<0.01$ (Student's *t* test)
[j]Different from group 3; $P<0.05$ (Student's *t* test)
[k]Different from group 3; $P<0.001$ (Student's *t* test)
[l]Different from group 5; $P<0.005$ ($\chi^2$ analysis)
[m]Different from group 5; $P<0.001$ (Student's *t* test)

**Figure 1** Cumulative incidences of MT in castrated male WF rats given DES alone (group 3) (△); DES and antiE (group 4) (▲); DES and NBU (group 5) (○); and DES, antiE, and NBU (group 6) (●).

**Figure 2** Effect of antiE on number and size of AC in castrated male WF rats given DES alone or in combination with NBU. Rectangles in upper histogram are those for rats given DES alone (▨), or DES and antiE (□), and rectangles on lower histogram are for NBU-treated rats given DES (□), or DES and antiE (▨).

in antiE-treated rats receiving DES and NBU compared with that in control rats without antiE (Table I). MT produced in group 6 were 5 adenocarcinomas (AC) and 3 fibroadenomas (FA). Therefore, the mean number of AC/rat was $0.3 \pm 0.1$. The mean number of MT/rat with MT was not different between groups 5 and 6.

As indicated in the distribution of AC with diameters >2 mm, most AC were observed as multiple nodules of various sizes (Figure 2). Compared to group 5, the number of AC in group 6 was significantly reduced in each size category, especially in that below 10 mm diameter. In group 4 no AC >10 mm was present. Therefore, antiE treatment affected the growth of MT as well as the development of MT.

*Body and pituitary weights*

The mean body weight was significantly reduced in castrated male rats treated with NBU alone or DES alone ($203 \pm 5$ g, $178 \pm 10$ g), compared with castrated control rats ($260 \pm 13$ g). On the other hand, there were no differences between antiE-treated rats given DES alone or in combination with NBU ($177 \pm 6$ g, $162 \pm 6$ g) and each corresponding control rat without antiE treatment ($178 \pm 10$ g, $165 \pm 165 \pm 5$ g). AntiE treatment did not further affect the DES-induced decrease in the body weight.

The relative pituitary weight and its distribution in each size are shown in Figure 3. The mean pituitary weight was reduced in groups 4 and 6 by approximately 50 and 80% compared to those in groups 3 and 5, respectively. The weight decreases by antiE treatment were similar to those found in castrated rats or castrated rats given NBU alone. AntiE treatment therefore, reveresed the increase in the pituitary weight by DES treatment. All the pituitary weights, except one case, were below 10 mg per 100 g body weight in group 4. None of the pituitary glands that weighed over 20 mg per 100 g body weight were observed in group 6. The reduction in the pituitary weight associated with antiE treatment was related to the fall in the occurrence of MT.

*Histology of pituitary gland*

Most PT was usually haemorragic chromophobeadenomas in histologic classification. Some cases of PT which weighed >30 mg were hyperplastic. The pituitary gland containing microadenoma was observed in an antiE-treated rat receiving DES alone or in combination with NBU. The appearance of the latter followed 2 AC and 2 FA in the mammary glands.

**Figure 3** Distribution of pituitary weights of castrated male WF rats treated variously: tumorous, ●; non-tumorous, ○: rats with MT, *.

### Discussion

We have shown in this study that the concurrent development of MT and PT induced by DES alone or in combination with NBU was markedly and concomitantly prevented when antiE treatment was administered. N-nitrosobutylurea compounds have been shown to be carcinogenic in the mammary gland in several strains of female rats (Gullino *et al.*, 1975; Odashima, 1970). In this study the amount of NBU was a subthreshold dose which could not induce MT in castrated male rats or female rats (Yokoro *et al.*, 1977). In contrast, the combination treatment of NBU and DES significantly increased the development of MT but not PT. These findings indicate that NBU and DES act synergistically in the induction of MT in castrated male rats. The anti-tumour effect of antiE on the mammary gland could be more clearly detected in rats given the combination of DES and NBU than in rats given DES alone. Sharp changes in the mean number of MT/rat and the number of MT in small to large sizes were also observed in those rats given the combination treatment. Therefore, antiE treatment inhibited the growth of MT as well as the development of MT.

AntiE has been thought to mimic oestrogenic action in binding to receptor sites on the oestrogen target tissues such as the uterus, vagina, and mammary tumour, and to prevent oestrogen from expressing its full effect on these tissues (Katzenellenbogen *et al.*, 1979; Kurl & Borthwick, 1980). Meanwhile, oestrogen stimulates prolactin secretion *in vivo* (Chen & Meites, 1970; Gala & Boss, 1975) and *in vitro* (Lu *et al.*, 1971; Nicoll & Meites, 1962). The pituitary gland and prolactin-secreting pituitary tumour bind oestradiol (Kato *et al.*, 1968; Noteboom *et al.*, 1982; Notides, 1970). In this study a close parallel correlation between antiE treatment and anti-oestrogenic events in the mammary and the pituitary glands could be seen. AntiE treatment was effective in reducing the incidence of PT and the pituitary weight. Although serum prolactin levels were not measured in this study, we have recently shown that there is a positive association between the DES-mediated prolactin levels and PT and MT development. Thus, the anti-MT effect of antiE was considered to be due to the declining serum prolactin levels caused by the prevention of DES-induced PT formation. This is also supported by recent findings of DeQuijida *et al.*, (1980) that the antiE compound, tamoxifen, has an inhibitory effect on the growth of transplantable prolactin-secreting rat pituitary tumour 7315 and its effect was accompanied by a decrease in the level of circulating prolactin. In contrast, a lack of inhibitory effect of antiE on oestrogen-stimulated prolactin secretion has been reported (Jordan & Koerner 1976). However, Kurl & Morris (1978)

showed differential depletion of cytoplasmic high affinity oestrogen receptors after *in vivo* treatment with clomiphene and tamoxifen, suggesting different penetration capabilities. Thus, this discrepancy may be related to differences in such capabilities among antiE compounds or to variables such as dose, mode and period of administration. AntiE might have also contributed by directly impairing oestrogen receptors in the mammary gland so as to render the tissue insensitive to DES stimuli, but this seems not to be the primary effect in this study.

We have provided evidence in the present and previous studies (Sumi *et al.*, 1983) that the induction of MT and PT can be controlled in rats under the influence of oestrogen stimuli by prolonged treatment with the dopamine agonist 2-bromoergocryptine (CB-154), or with the oestrogen antagonist, clomiphene citrate. Although it may be difficult to define the relative efficiency of these treatments on anti-MT, both treatments applied to rats given DES and NBU exhibited a similar effect on the pituitary gland. However, a few differences were observed. Since MT produced in CB-154-treated and castrated rats that had been given DES and NBU were mostly FA, AC incidence was only 1/20 (5%) (Sumi *et al.*, 1983), as opposed to 4/17 (24%) in antiE-treated rats given DES and NBU. Moreover, the onset and the mean latency of MT in antiE-treated group were ~5 months earlier and 3 months shorter, respectively, in spite of ~1.5 months earlier commencement of antiE treatment than CB-154 treatment. Thus, CB-154 appears to be more efficient in anti-mammary tumorigenesis than antiE. The lack of appreciable effect on the early onset of MT or the lack of complete blockade of AC may be relevant to the property of antiE that can exhibit both antagonistic and agonistic actions (Bowman *et al.*, 1981; Katzellenbogen *et al.*, 1979). Oestrogen produces a direct stimulatory effect and indirectly stimulates DNA synthesis of mammary epithelial cells through hypersecretion of prolactin (Nagasawa *et al.*, 1976). Both actions thus promote the interaction of the target tissues to carcinogens. As shown in our previous study (Sumi *et al.*, 1980), DES treatment is a prerequisite for male mammary tumorigenesis, by feminizing the mammary gland of castrated male rats. Therefore, antagonistic effect of antiE might have been insufficient during an earlier phase when DES treatment conceivably acts to render cells more susceptible to NBU treatment.

This work has been supported in part by a Grant-in-Aid for Encouragement of Young Scientists, and for Scientific Research from the Ministry of Education, Science and Culture, Japan.

We express our gratitude to Dr. D.H. Nelson and Dr. D.K. Murray, University of Utah (Utah, U.S.A.) and Dr. K. Matsumoto, Osaka University (Osaka, Japan) for stimulating discussions. We thank Mr. T. Nishioka, Miss M. Sasaki, Mr. K.E. Johnson and Mr. H.N. Sekiya for their excellent technical assistance.

### References

BOWMAN, S.P., LEAKE, A. MILLER, M. & MORRIS, I.D. (1981). Agonist and antagonist activities of enclomiphene upon oestrogen-mediated events in the uterus, pituitary gland and brain of the rat. *J. Endocrinol.*, **88**, 367.

CHEN, C.L. & MEITES, J. (1970). Effect of estrogen and progesterone on serum and pituitary prolactin levels in ovariectomized rats. *Endocrinology*, **86**, 503.

CLIFTON, K.H. & MEYER, R.K. (1956). Mechanism of anterior pituitary tumor induction by estrogen. *Anat. Rec.*, **125**, 65.

DEQUIJIDA, M., TIMMERMANS, H.A.T. & LAMBERTS, S.W.J. (1980). Tamoxifen suppresses both the growth of prolactin-secreting pituitary tumors and normal prolactin synthesis in the rats. *J. Endocrinol.*, **86**, 109.

GALA, R.R. & BOSS, R.S. (1975). Serum prolactin levels of rats under continuous estrogen stimulation and 2-Br-α-ergocryptine (CB-154) injection. *Proc. Soc. Exp. Biol. Med.*, **149**, 330.

GULLINO, P.M., PETTIGREW, H.M. & GRANTHAM, F.H. (1975). N-nitrosomethylurea as mammary gland carcinogen in rats. *J. Natl Cancer Inst.*, **54**, 401.

HEUSON, J.C., ENGELSMAN, E., BLONK-VAN DEL WIJIST, J. & 5 others. (1975). Comparative trial of nafoxidine and ethinyloestradiol in advanced breast cancer: An E.O.R.T.C. study. *Br. Med. J.*, **ii**, 711.

JORDAN, V.C. & KOERNER, S. (1976). Tamoxifen as an anti-tumor agent: Role of estradiol and prolactin. *J. Endocrinol.*, **68**, 305.

KATO, J., KOBAYASHI, T. & VILLEE, C.A. (1968). Effect of clomiphene on the uptake of estradiol by the anterior hypothalamus and hypophysis. *Endocrinology*, **82**, 1049.

KATZENELLENBOGEN, B.S., TSAI, T.S., TATEE, T. & KATZENELLENBOGEN, J.A. (1979). Estrogen and antiestrogen action: studies in reproductive target tissues and tumors. *Adv. Exp. Med. Biol.*, **177**, 111.

KURL, R.N. & BORTHWICK, N.M. (1980). Clomiphene and tamoxifen action in the rat uterus. *J. Endocrionol.*, **85**, 519.

KURL, R.N. & MORRIS, I.D. (1978). Differential depletion of cytoplasmic high affinity oestrogen receptors after the *in vivo* adminstration of the antiestrogens, clomiphene, MER-25 and tamoxifen. *Br. J. Pharmacol.*, **62**, 487.

LAMBERTS, S.W.J., VERLEUN, T. & OOSTEROM, R. (1982). Effect of tamoxifen administration on prolactin release by invasive prolactin-secreting pituitary adenomas. *Neuroendocrinology.*, **34**, 339.

LU, K.H., KOCH, Y. & MEITES, J. (1971). Direct inhibition by ergocornine of pitutiary prolactin release. *Endocrinology*, **89**, 229.

NAGASAWA, H., YANAI, R. & TANGIGUCHI, H. (1976). Importance of mammary gland DNA synthesis on carcinogen-induced mammary tumorigenesis in rats. *Cancer Res.*, **36**, 2223.

NICOLL, C.S. & MEITES, J. (1962). Estrogen stimulation of prolactin production by rat adenohypophysis *in vitro*. *Endocrinology*, **70**, 272.

NOTEBOOM, W.D., DURHAM, J.B. & MITRA, R. (1982). Variations in the levels of estrogen receptors in prolactin producing pituitary tumor cells. *J. Steroid Biochem.*, **16**, 633.

NOTIDES, A.C. (1970). Binding affinity and specificity of the estrogen receptor of the rat uterus and anterior pituitary. *Endocrinology.*, **87**, 987.

ODASHIMA, S. (1970). Leukemogenesis of N-nitrosobutylurea in the rats. 1. Effect of various concentrations in the drinking water to female donryu rats. *Gann*, **61**, 245.

SUMI, C., YOKORO, K., KAJITANI, T. & ITO, A. (1980). Synergism of diethylstilbestrol and other carcinogens in concurrent development of hepatic, mammary, and pituitary tumors in castrated male rats. *J. Natl Cancer Inst.*, **65**, 169.

SUMI, C., YOKORO, K. & MATSUSHIMA, R. (1983). Suppression of diethylstilbestrol and N-nitrosobutylurea-induced mammary and pituitary tumorigenesis in rats by prolonged treatment with 2-bromoergocryptine. *Cancer Res.*, **43**, 4781.

YOKORO, K., NAKANO, M., ITO, NAGAO, K., KODAMA, Y. & HAMADA, K. (1977). Role of prolactin in rat mammary carcinogenesis: Detection of carcinogenicity of low-dose carcinogens and of persisting dormant cancer cells. *J. Natl Cancer Inst.*, **54**, 401.